# Menstrual Cycle Regularity and Length Across the Reproductive Lifespan and Risk of Cardiovascular Disease

**DOI:** 10.1001/jamanetworkopen.2022.38513

**Published:** 2022-10-25

**Authors:** Yi-Xin Wang, Jennifer J. Stuart, Janet W. Rich-Edwards, Stacey A. Missmer, Kathryn M. Rexrode, Leslie V. Farland, Kenneth J. Mukamal, Scott M. Nelson, Caren G. Solomon, Abigail Fraser, Jorge E. Chavarro

**Affiliations:** 1 Department of Nutrition, Harvard T.H. Chan School of Public Health, Boston, Massachusetts; 2 Department of Environmental Health, Harvard T.H. Chan School of Public Health, Boston, Massachusetts; 3 Division of Women’s Health, Department of Medicine, Brigham and Women’s Hospital and Harvard Medical School, Boston, Massachusetts; 4 Department of Epidemiology, Harvard T.H. Chan School of Public Health, Boston, Massachusetts; 5 Department of Obstetrics, Gynecology, and Reproductive Biology, College of Human Medicine, Michigan State University, Grand Rapids; 6 Department of Epidemiology and Biostatistics, Mel and Enid Zuckerman College of Public Health, Tucson, Arizona; 7 Division of General Medicine, Beth Israel Deaconess Medical Center and Harvard Medical School, Boston, Massachusetts; 8 School of Medicine, University of Glasgow, Glasgow, United Kingdom; 9 Department of Population Health Sciences, Bristol Medical School, University of Bristol, Bristol, United Kingdom; 10 Channing Division of Network Medicine, Department of Medicine, Brigham and Women’s Hospital and Harvard Medical School, Boston, Massachusetts

## Abstract

**Importance:**

Menstrual cycle characteristics may be associated with an increased risk of cardiovascular disease (CVD). However, existing studies are limited, and few have explored the mediating role of established CVD risk factors.

**Objective:**

To explore the associations of menstrual cycle characteristics across the reproductive lifespan with the risk of CVD and to what extent these associations were mediated by hypercholesterolemia, chronic hypertension, and type 2 diabetes.

**Design, Setting, and Participants:**

This cohort study prospectively followed Nurses’ Health Study II participants between 1993 and 2017 who reported menstrual cycle regularity and length for ages 14 to 17 years and 18 to 22 years at enrollment in 1989 and updated current cycle characteristics in 1993 (at ages 29 to 46 years). Data analysis was performed from October 1, 2019, to January 1, 2022.

**Exposures:**

Menstrual cycle regularity and length across the reproductive lifespan.

**Main Outcomes and Measures:**

Incident CVD events of interest, including fatal and nonfatal coronary heart disease (CHD; myocardial infarction [MI] or coronary revascularization) and stroke.

**Results:**

A total of 80 630 Nurses’ Health Study II participants were included in the analysis, with a mean (SD) age of 37.7 (4.6) years and body mass index of 25.1 (5.6) at baseline. Over 24 years of prospective follow-up, 1816 women developed their first CVD event. Multivariable Cox proportional hazards models showed that, compared with women reporting very regular cycles at the same ages, women who had irregular cycles or no periods at ages 14 to 17, 18 to 22, or 29 to 46 years had hazard ratios for CVD of 1.15 (95% CI, 0.99-1.34), 1.36 (95% CI, 1.06-1.75), and 1.40 (95% CI, 1.14-1.71), respectively. Similarly, compared with women reporting a cycle length of 26 to 31 days, women reporting a cycle length 40 days or more or a cycle too irregular to estimate from ages 18 to 22 or 29 to 46 years had hazard ratios for CVD of 1.44 (95% CI, 1.13-1.84) and 1.30 (95% CI, 1.09-1.57), respectively. Mediation analyses showed that subsequent development of hypercholesteremia, chronic hypertension, and type 2 diabetes only explained 5.4% to 13.5% of the observed associations.

**Conclusions and Relevance:**

In this cohort study, both irregular and long menstrual cycles were associated with increased rates of CVD, which persisted even after accounting for subsequently established CVD risk factors.

## Key Points

**Question:**

Are menstrual cycle characteristics across the reproductive lifespan associated with the risk of cardiovascular disease (CVD), and to what extent are these associations mediated by hypercholesterolemia, chronic hypertension, and diabetes?

**Findings:**

In this cohort study of 80 630 women, irregular and long menstrual cycles across the reproductive lifespan were associated with an increased risk of CVD, independent of established cardiovascular disease risk factors. Mediation analysis showed that only a small proportion of the associations were explained by hypercholesterolemia, chronic hypertension, and type 2 diabetes.

**Meaning:**

These results suggest that menstrual cycle characteristics throughout the reproductive lifespan may be used as additional markers of CVD risk in women.

## Teaser

This cohort study of US nurses examines the association of irregular and long menstrual cycles across the reproductive lifespan with risk of cardiovascular disease.

## Introduction

There is growing recognition that female reproductive characteristics, particularly those that manifest earlier in life and before the onset of established cardiovascular disease (CVD) risk factors, may serve as informative markers of future CVD risk for women.^1,2,3,4,5^ The American College of Obstetricians and Gynecologists endorses consideration of menstrual cycle characteristics as an additional vital sign beginning in adolescence, reinforcing its importance in health assessments and its connection to overall health status.^6^ To date, several studies have reported increased risks of CVD morbidity or mortality in women with irregular cycles in early or mid-adulthood.^4,7,8,9^ However, the associations of irregular and long cycles at different points throughout a woman’s reproductive lifespan with CVD remain unclear. Furthermore, while growing evidence shows that irregular and long cycles are associated with a higher risk of conditions that are well-established CVD risk factors, such as hypercholesterolemia,^10^ chronic hypertension,^10^ and type 2 diabetes,^11^ limited data are available to inform the extent to which these may mediate the associations between cycle characteristics and CVD risk.

We therefore estimated associations of menstrual cycle regularity and length at different age ranges (14 to 17, 18 to 22, and 29 to 46 years) with subsequent CVD events among women in the Nurses’ Health Study II (NHS II). We also explored to what extent these associations were mediated by the subsequent development of hypercholesterolemia, chronic hypertension, and type 2 diabetes.

## Methods

### Study Population

The NHS II is an ongoing prospective cohort of 116 429 female registered nurses in the US who were enrolled in 1989 at ages 25 to 42 years. Participants have been followed every 2 years through questionnaires that collect information on demographic and behavioral characteristics, reproductive factors, and health outcomes. Return of completed questionnaires indicated participants’ written informed consent. Study procedures have been approved by the institutional review boards of the Brigham and Women’s Hospital and Harvard T.H. Chan School of Public Health. The present study was reported according to the Strengthening the Reporting of Observational Studies in Epidemiology (STROBE) guidelines.

### Menstrual Cycle Characteristics

On the 1989 enrollment questionnaire, participants reported usual patterns of menstrual cycle regularity and length, excluding during pregnancy, lactation, or when using oral contraceptives, at 2 time periods: during high school (ie, ages 14 to 17 years) and between the ages of 18 and 22 years. On the 1993 biennial questionnaire, participants reported their current usual cycle patterns (ages 29 to 46 years). Cycle regularity was categorized as very regular (no more than 3 to 4 days before or after expected), regular (within 5 to 7 days), usually irregular, always irregular, or no periods. Usual cycle length, defined as the interval from the first day of the period to the first day of the following period, was reported as under 21 days, 21 to 25 days, 26 to 31 days, 32 to 39 days, 40 to 50 days, or more than 50 days or too irregular to estimate. Self-report of cycle characteristics has been shown to be reliable in previous NHS II and other studies.^11,12,13^

### Cardiovascular Disease Ascertainment

Incident CVD events of interest included fatal and nonfatal coronary heart disease (CHD; myocardial infarction or coronary revascularization, including coronary artery bypass graft surgery or percutaneous coronary intervention) and stroke, which were identified based on nurse participant self-report on biennial questionnaires and confirmed by medical record review.^2^ Coronary revascularization procedures were self-reported, which has been previously validated in a subgroup of participants from the Health Professionals Follow-up Study.^14^ Deaths were identified by next of kin and postal authorities, or through a search of the National Death Index; over 98% of NHS II deaths were able to be ascertained.^15^

### Assessment of Covariates

Participants self-reported race and ethnicity, age at menarche, and height at NHS II enrollment in 1989. Weight, parental history of CVD (myocardial infarction or stroke) before age 60 years, reproductive characteristics, and behavioral factors were ascertained every 2 to 4 years over follow-up. Body mass index (BMI; calculated as weight in kilograms divided by height in meters squared) was calculated based on baseline height and updated weight for every follow-up cycle. Physical activity was reported in 1989, 1991, 1997, 2001, 2005, 2009, and 2013. Diet was assessed every 4 years beginning in 1991 using a validated semiquantitative food frequency questionnaire, which was used to calculate the 2010 Alternative Healthy Eating Index (AHEI). As described elsewhere,^4,11,16^ AHEI-2010 summarizes overall diet quality based on 11 components; the total score ranges from 0 to 110, with a higher score indicating a healthier diet. Most of the self-reported covariates have been validated or shown to be reliable in a subgroup of participants from this cohort or the original NHS cohort.^17,18,19^ Hypercholesterolemia, chronic hypertension, and type 2 diabetes were self-reported in 1989 with biennial questionnaires from 1991 forward capturing incident diagnoses. Previous NHS or NHS II medical record validation studies confirmed 86%, 98%, and 94% of self-reported hypercholesterolemia, type 2 diabetes, and chronic hypertension, respectively.^18,20,21^

### Exclusions

We excluded participants who had died or developed CVD (1250 participants) or reached menopause before 1993 (5310 participants), opted out during follow-up (16 participants) or never returned follow-up questionnaires (893 participants), or had missing data on cycle characteristics for 1 or more of the age ranges of interest (28 330 participants) (eFigure in the Supplement). Baseline age-standardized characteristics were similar between participants included in the analysis (80 630 participants) and those excluded due to incomplete data on cycle characteristics (28 330 participants) (eTable 1 in the Supplement). Women who used oral contraceptives at baseline (9899 participants) and those who developed hypercholesterolemia (11 858 participants), chronic hypertension (4810 participants), or type 2 diabetes (205 participants) before 1993 were additionally excluded from the mediation analyses (eFigure in the Supplement).

### Statistical Analysis

Participants’ person-years of follow-up were calculated from the date of 1993 questionnaire return until the date of CVD event, death, last returned questionnaire, or end of follow-up (June 30, 2017), whichever occurred first. We used Cox proportional hazards models with time-varying age and calendar time (in 2-year intervals) as the underlying timescale to separately estimate hazard ratios (HR) and 95% CIs for the associations of cycle regularity and length at different age ranges (14 to 17, 18 to 22, or 29 to 46 years) with CVD. To reduce potential exposure misclassification, women who used oral contraceptives for more than 2 months per year during the age ranges under study were included in a separate exposure category, because oral contraceptives may be used as a treatment for common ovulation disorders and affect cycle characteristics.^22^ Analyses were conducted using SAS version 9.4 (SAS Institute Inc) and statistical significance was set at a 2-tailed *P* < .05.

We included the following covariates as potential confounders: age at menarche (continuous), race and ethnicity (White, Black, Hispanic, or Asian), parental history of CVD before age 60 years (yes, no), baseline BMI (below 23, 23 to 24.9, 25 to 29.9, 30 to 34.9, or 35 and above), and time-varying menopausal status and hormone usage (premenopausal, postmenopausal and never hormone therapy use, postmenopausal and past hormone therapy use, or current hormone therapy use), parity (1 or below, 2, or 3 or more births), and regular aspirin use (yes, no). Because behavioral factors are important determinants of CVD risk and may directly affect some of the underlying metabolic disturbances associated with menstrual cycle disorders,^4^ we additionally adjusted for time-varying physical activity (0, 0.1 to 1.0, 1.1 to 3.4, 3.5 to 5.9, or 6 or more h/wk), smoking status (never, former, current smoker [further categorized as 1 to 14, 15 to 24, or 25 or more cigarettes/d), and AHEI diet quality score (quintiles). To address missing confounder data, covariate values were carried forward for one cycle after which a missing indicator variable was included in the model, which has been shown to induce minimal bias.^23^

To assess the role of persistent cycle irregularity or atypical patterns over time, we jointly examined information on participants’ cycle characteristics across different age ranges; we estimated the risk of CVD according to joint exposure categories of cycle regularity and length at ages 18 to 22 years and 29 to 46 years. We also investigated the interaction between cycle dysfunction and behavioral factors (smoking, diet quality, and physical activity) and BMI on CVD risk both on the multiplicative and additive scales.^11,24^ Finally, we conducted a mediation analysis to examine what proportion of the observed association between cycle characteristics (at ages 18 to 22 and 29 to 46 years) and CVD was accounted for by hypercholesterolemia, chronic hypertension, and type 2 diabetes.^25,26^

## Results

The 80 630 NHS II participants included in the analysis had a mean (SD) age of 37.7 (4.6) years and BMI of 25.1 (5.6) at baseline. Irregular (ie, “usually or always irregular” or no periods) and long cycles (32 or more days or too irregular to estimate) were reported by 7366 (9.1%) and 11 199 participants (13.9%) aged 29 to 46 years. Compared with women reporting regular cycles, women who experienced irregular cycles had a higher mean BMI (mean [SD] BMI, 27.9 [7.7] vs 25.0 [5.4]) and were more likely to have hypercholesterolemia (23.5% [847 of 3572 participants] vs 15.3% [7384 of 47 632 participants]) and chronic hypertension (12.5% [449 of 3572 participants] vs 6.5% [3169 of 47 632 participants]) at baseline (Table 1). A similar pattern of difference was observed between women reporting cycle lengths of 26 to 36 days and long cycles.

**Table 1.** Age-Standardized Study Population Characteristics by Menstrual Cycle Regularity and Length Between Ages 29 and 46 Years

Characteristics^a^^,^^b^	Participants, No. (%)								
	Cycle regularity				Cycle length				Oral contraceptive users (n = 9899)
	Very regular (n = 44 271)	Regular (n = 19 094)	Usually irregular (n = 4625)	Always irregular/no period (n = 2741)	≤25 d (n = 11 900)	26-31 d (n = 47 632)	32-39 d (n = 7627)	≥40 d/too irregular to estimate (n = 3572)	
Age, mean (SD), y	38.1 (4.4)	38.3 (4.5)	38.5 (4.9)	37.7 (4.9)	39.4 (4.2)	38.1 (4.4)	36.8 (4.4)	37.9 (5.1)	34.7 (4.1)
Age at menarche, mean (SD), y	12.4 (1.4)	12.5 (1.4)	12.5 (1.6)	12.6 (1.6)	12.3 (1.4)	12.4 (1.4)	12.6 (1.5)	12.6 (1.6)	12.5 (1.4)
Race or ethnicity									
White	42 505 (96.0)	18 177 (95.2)	4370 (94.4)	2601 (94.9)	11 300 (95.0)	45 670 (95.9)	7308 (95.7)	3375 (94.5)	9455 (95.3)
Non-White	1766 (4.0)	917 (4.8)	255 (5.6)	140 (5.1)	600 (5.0)	1962 (4.1)	319 (4.3)	197 (5.5)	444 (4.7)
Current smoker	4651 (10.5)	2089 (10.9)	543 (11.4)	321 (11.8)	1744 (14.9)	4864 (10.2)	614 (8.3)	382 (10.6)	838 (7.7)
Physical activity, mean (SD), h/wk	2.7 (3.8)	2.5 (3.6)	2.5 (3.8)	2.5 (4.1)	2.7 (4)	2.6 (3.8)	2.5 (3.6)	2.4 (3.7)	3.0 (4.2)
BMI, mean (SD)	25.0 (5.3)	25.1 (5.6)	26.6 (6.9)	28.2 (7.8)	24.8 (5.3)	25.0 (5.4)	26.1 (6.5)	27.9 (7.7)	24.3 (4.9)
Regular aspirin use	3728 (8.2)	1691 (8.5)	455 (9.3)	262 (9.6)	1170 (8.9)	4013 (8.2)	636 (9.0)	317 (8.9)	709 (9.8)
Alcohol consumption, mean (SD), g/d	3.2 (6.1)	2.9 (5.8)	2.7 (5.3)	2.6 (6.4)	3.0 (5.8)	3.2 (6.1)	2.7 (5.5)	2.5 (5.6)	3.7 (6.0)
Hypercholesterolemia	6672 (14.9)	3237 (16.6)	959 (20.1)	665 (24.3)	1941 (15.4)	7384 (15.3)	1361 (18.9)	847 (23.5)	1807 (20.2)
Chronic hypertension	2803 (6.2)	1419 (7.1)	527 (11.1)	368 (13.4)	919 (7.1)	3169 (6.5)	580 (8.3)	449 (12.5)	420 (5.4)
Type 2 diabetes	93 (0.2)	74 (0.4)	23 (0.5)	26 (1.0)	29 (0.2)	127 (0.3)	32 (0.5)	28 (0.8)	16 (0.2)
AHEI score, mean (SD)^c^	48.1 (10.8)	47.5 (10.7)	47.3 (10.7)	46.8 (10.8)	47.8 (10.8)	47.9 (10.8)	47.6 (10.8)	47.1 (10.7)	48.3 (11.0)
Lowest quintile (unhealthy)	7620 (17.4)	3534 (18.9)	887 (19.5)	565 (20.6)	2015 (17.8)	8409 (17.9)	1474 (18.9)	708 (19.9)	1880 (16.9)
Highest quintile (healthy)	8277 (18.4)	3378 (17.3)	786 (16.6)	432 (15.7)	2223 (17.8)	8746 (18.1)	1326 (18.0)	578 (15.9)	1618 (18.8)
Parity, mean (SD)	1.7 (1.2)	1.8 (1.2)	1.6 (1.2)	1.6 (1.2)	1.7 (1.2)	1.7 (1.2)	1.8 (1.2)	1.6 (1.3)	1.3 (1.2)
Parental history of CVD before age 60 y^b^	7323 (16.4)	3237 (16.7)	852 (18.2)	504 (18.6)	2145 (17.6)	7845 (16.3)	1285 (17.3)	641 (18.1)	1417 (15.7)

Abbreviations: AHEI, Alternative Healthy Eating Index; BMI, body mass index (calculated as weight in kilograms divided by height in meters squared); CVD, cardiovascular disease.

^a^ Means (SD) for continuous variables and No. (%) for categorical variables are standardized to the age distribution of the study population, except for age.

^b^ A total of 108 (0.1%), 343 (0.4%), 116 (0.1%), 1150 (1.4%), and 8198 (10.2%) women had missing data on baseline smoking status, physical activity, BMI, aspirin use, and diet (including alcohol intake), respectively.

^c^ The AHEI-2010 (Alternative Healthy Eating Index) score ranges from 0 (nonadherence) to 110 (perfect adherence) with a higher score indicating a healthier diet.

**Figure 1.**
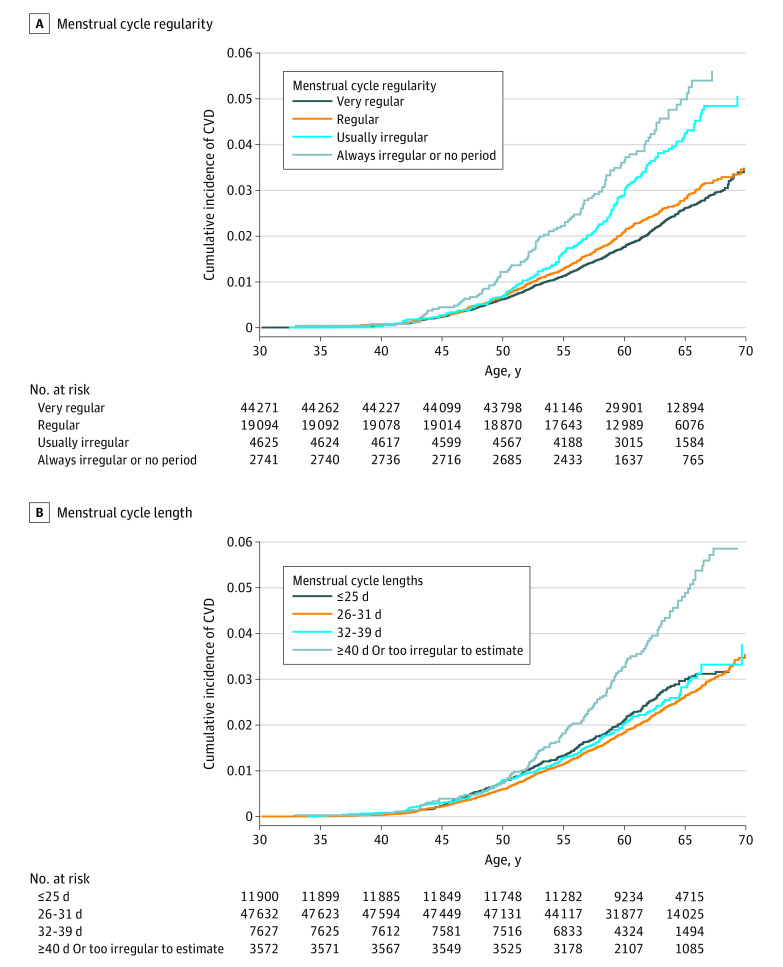
Incidence of Cardiovascular Disease (CVD) According to Menstrual Cycle Regularity and Length Between Ages 29 and 46 Years

**Figure 2.**
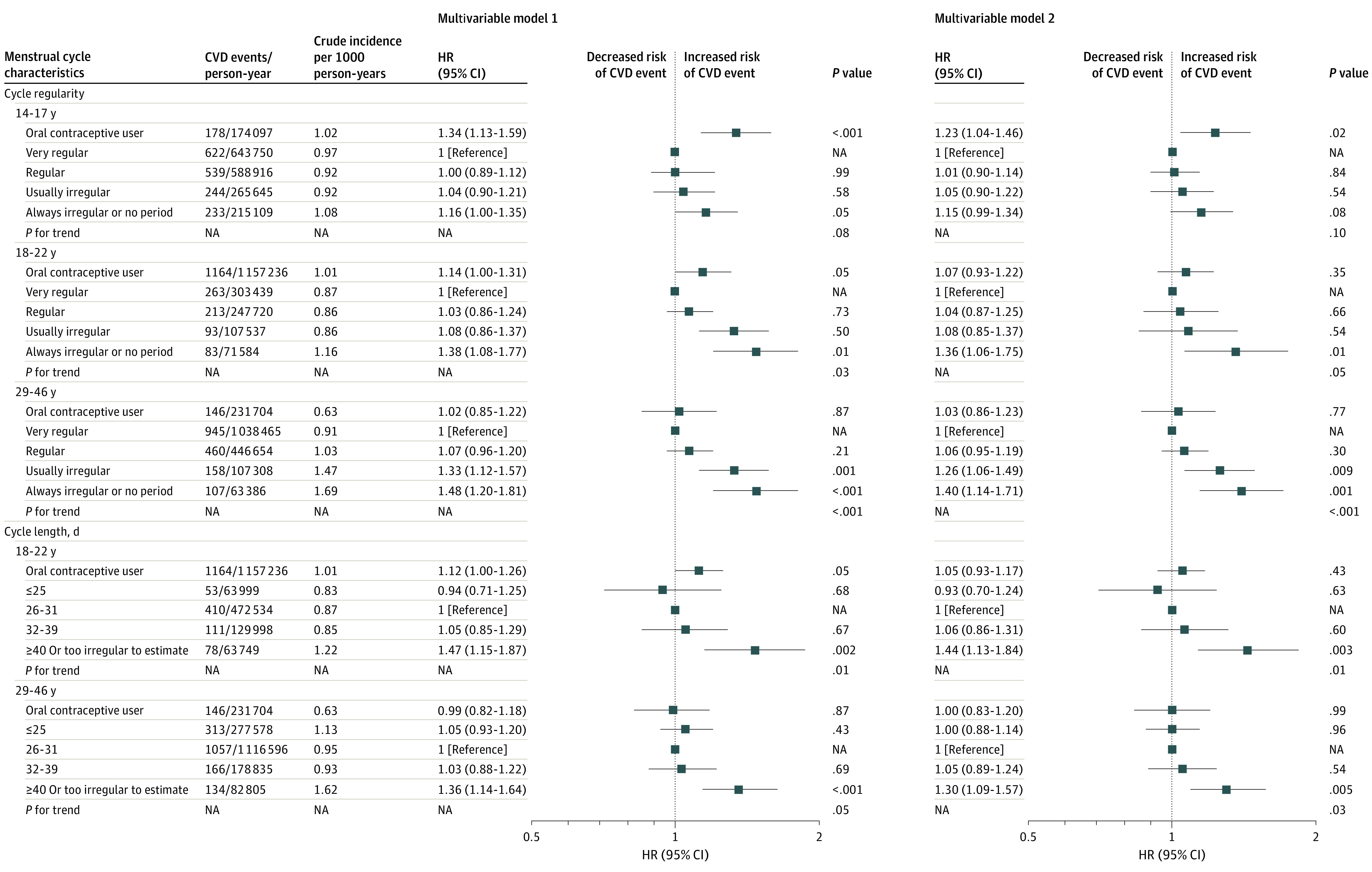
Hazard Ratios (HRs) for Cardiovascular Disease (CVD) Events According to Menstrual Cycle Regularity and Length NA indicates not applicable. Model 1 was adjusted for age (continuous), age at menarche (continuous), race/ethnicity (White [reference], Black, Hispanic, Asian), parental history of CVD before age 60 years (yes, no), baseline body mass index (<23 [reference], 23-24.9, 25-29.9, 30-34.9, ≥35), and time-varying menopausal status and hormone usage (premenopausal [reference], postmenopausal and never hormone therapy use, postmenopausal and past hormone therapy use, or current hormone therapy use), parity (≤1 [reference], 2, ≥3 births), and regular aspirin use (yes, no [reference]). Model 2 was additionally adjusted for time-varying physical activity (0 [reference], 0.1-1.0, 1.1-3.4, 3.5-5.9, ≥6 hours/week), smoking status (never smoker [reference], former smoker, current smoker: 1-14, 15-24, ≥25 cigarettes/d), and Alternative Healthy Eating Index diet quality score (quintiles, with the lowest quintile [reference] representing the least healthy diet). The *P* value for trends was estimated by excluding women using oral contraceptives.

Over 24 years (1 887 517 person-years) of prospective follow-up, 1816 women (2.3%) developed an incident CVD event (CHD, 1193 women [1.5%]; stroke, 636 women [0.8%]). The crude cumulative incidence of CVD after age 50 years was both higher among women who reported irregular vs regular cycles, and cycle lengths of 40 days or more or too irregular to estimate vs 39 days or fewer at ages 29 to 46 years (Figure 1). Women who reported always irregular or no periods from 14 to 17 years of age had an HR for CVD of 1.16 (95% CI, 1.00-1.35) compared with women with very regular cycles at that age in partially adjusted models (Figure 2); results were not significant when adjusted for updated behavioral factors (HR, 1.15; 95% CI, 0.99-1.34). Analyses of cycle regularity and length at ages 18 to 22 years and 29 to 46 years demonstrated consistent trends with an increasing rate of CVD observed across categories of decreasing regularity and increasing cycle length. When comparing always irregular or no period with very regular cycles, ages 18 to 22 years had an HR of 1.36 (95% CI, 1.06-1.75) and ages 29 to 46 years an HR of 1.40 (95% CI, 1.14-1.71). When comparing cycles of 40 days or more or too irregular to estimate with 26 to 31 days, the HR for ages 18 to 22 years was 1.44 (95% CI, 1.13-1.84) and for ages 29 to 46 years, 1.30 (95% CI, 1.09-1.57) (Figure 2).

Women with usually irregular or always irregular or no periods and those with very long cycles had increased rates of CHD but not stroke at ages 29 to 46 years (eTable 2 in the Supplement). When cycle regularity and length were jointly classified, the highest rate of CVD was observed among women reporting both irregular and long cycles (32 days or more) at ages 18 to 22 and 29 to 46 years (eTable 3 in the Supplement); however, there was no evidence of interaction between cycle regularity and length on either the multiplicative or additive scales. We observed a positive additive interaction (ie, relative excess risk due to interaction [RERI] above 0) between irregular cycles at ages 29 to 46 years and having overweight or obesity (ie, BMI of 25 or greater) on the rate of CVD (RERI, 0.55; 95% CI, 0.10-1.01) (eTable 4 in the Supplement). However, there was no strong evidence of multiplicative or additive interaction between cycle characteristics and smoking, diet quality, or physical activity. When women were cross-classified according to cycle regularity from ages 14 to 17 and 29 to 46 years, the rate of CVD was highest among women whose cycle changed from regular to irregular (HR, 1.34; 95% CI, 1.11-1.63) (Table 2). When we analyzed the change in cycle characteristics between 18 to 22 years and 29 to 46 years, the highest rate of CVD was observed among women who experienced persistent irregular or long cycles.

**Table 2.** Multivariable Adjusted Hazard Ratios (HR) for Cardiovascular Disease (CVD) Events According to Changes in Menstrual Cycle Characteristics

Changes in menstrual cycle characteristics	CVD events	Person-years	Multivariable adjusted HR (95% CI)	
			Model 1^a^	Model 2^b^
**Cycle regularity from ages 14-17 to 29-46 y^c^**				
Regular (no change)	946	1 027 827	1 [Reference]	1 [Reference]
Regular to irregular	120	63 838	1.45 (1.19-1.75)	1.34 (1.11-1.63)
Irregular to regular	335	329 119	1.14 (1.01-1.29)	1.13 (0.99-1.28)
Irregular (no change)	111	89 361	1.28 (1.05-1.56)	1.24 (1.01-1.51)
Oral contraceptive use at either age	304	377 373	1.22 (1.06-1.39)	1.17 (1.02-1.34)
**Cycle regularity from ages 18-22 to 29-46 y^c^**				
Regular (no change)	404	470 311	1 [Reference]	1 [Reference]
Regular to irregular	39	24 825	1.17 (0.84-1.63)	1.08 (0.78-1.50)
Irregular to regular	109	116 368	1.12 (0.91-1.39)	1.10 (0.89-1.36)
Irregular (no change)	53	40 461	1.49 (1.12-1.99)	1.46 (1.09-1.95)
Oral contraceptive use at either age	1211	1 235 551	1.14 (1.01-1.27)	1.06 (0.94-1.18)
**Cycle length from ages 18-22 to 29-46 y**				
<32 d (no change)	385	442 748	1 [Reference]	1 [Reference]
<32 d to ≥32 d	41	36 700	1.03 (0.75-1.43)	1.00 (0.73-1.39)
≥32 d to <32 d	105	101 671	1.18 (0.95-1.46)	1.17 (0.94-1.45)
≥32 d (no change)	74	70 847	1.35 (1.05-1.73)	1.36 (1.06-1.75)
Oral contraceptive use at either age	1211	1 235 551	1.14 (1.01-1.28)	1.07 (0.95-1.20)

^a^ Model 1 was adjusted for age (continuous), age at menarche (continuous), race/ethnicity (White [reference], Black, Hispanic, Asian), parental history of CVD before age 60 (yes, no [reference]), baseline body mass index (<23 [reference], 23-24.9, 25-29.9, 30-34.9, ≥35), time-varying menopausal status and hormone usage (premenopausal [reference], postmenopausal and never hormone therapy use, postmenopausal and past hormone therapy use, or current hormone therapy use), parity (≤1 [reference], 2, ≥3 births), and regular aspirin use (yes, no [reference]).

^b^ Model 2 was further adjusted for time-varying physical activity (0 [reference], 0.1-1.0, 1.1-3.4, 3.5-5.9, ≥6 hours/week), smoking status (never smoker [reference], former smoker, current smoker: 1-14, 15-24, ≥25 cigarettes/d), and Alternative Healthy Eating Index diet quality score (quintiles, with the lowest quintile [reference] representing the least healthy diet).

^c^ The analysis of cycle regularity over time considered “very regular” and “regular” as regular, and “usually irregular” and “always irregular/no period” as irregular.

Approximately 14% of the association between cycle irregularity at ages 18 to 22 years and incident CVD was jointly accounted for by the subsequent development of hypercholesterolemia, chronic hypertension, and type 2 diabetes (proportion mediated, 13.5%; 95% CI, 3.7%-38.8%) (Table 3). The proportion of the association between long cycle lengths at ages 18 to 22 years and CVD accounted for by established CVD risk factors was 9.0% (95% CI, 3.0%-24.3%). A slightly lower proportion of the associations between irregular (5.4%; 95% CI, 1.7%-16.0%) and long cycles (8.7%; 95% CI, 2.0%-31.4%) at ages 29 to 46 years with CVD were mediated by these established CVD risk factors.

**Table 3.** Multivariable Adjusted Hazard Ratios (HR) for Associations of Menstrual Cycle Regularity and Length With Cardiovascular Disease (CVD) Events^a^

Measure	CVD by cycle regularity, HR (95% CI)					CVD by cycle length, HR (95% CI)				
	Regular	Irregular/no period				<32 d	≥32 d			
		Ages 18-22 y	*P* value	Ages 29-46 y	*P* value		Ages 18-22 y	*P* value	Ages 29-46 y	*P* value
Estimation										
Without mediators (total effect)	1 [Reference]	1.16 (0.97-1.39)	NA	1.20 (0.99-1.47)	NA	1 [Reference]	1.18 (1.00-1.41)	NA	1.14 (0.95-1.36)	NA
With mediators (direct effect)	1 [Reference]	1.14 (0.95-1.36)	NA	1.19 (0.98-1.45)	NA	1 [Reference]	1.17 (0.98-1.38)	NA	1.13 (0.94-1.34)	NA
Proportion mediated, %^b^	NA	13.5 (3.7-38.8)	<.001	5.4 (1.7-16.0)	<.001	NA	9.0 (3.0-24.3)	<.001	8.7 (2.0-31.4)	<.001
Type 2 diabetes	NA	12.9 (3.3-39.0)	<.001	7.3 (3.5-14.5)	<.001	NA	14.0 (4.4-36.4)	<.001	15.0 (4.5-39.9)	<.001
Chronic hypertension	NA	10.0 (2.7-31.3)	<.001	2.6 (0.9-7.3)	.005	NA	9.7 (3.1-26.6)	<.001	3.9 (0.9-15.2)	.002
Hypercholesterolemia	NA	11.0 (3.0-33.1)	<.001	2.1 (0.8-5.6)	.003	NA	6.9 (2.2-19.4)	<.001	4.6 (1.4-13.8)	<.001

Abbreviation: NA, not applicable.

^a^ Models were adjusted for age (continuous), age at menarche (continuous), race or ethnicity (White [reference], Black, Hispanic, Asian), parental history of CVD before age 60 (yes, no [reference]), baseline body mass index (<23 [reference], 23-24.9, 25-29.9, 30-34.9, ≥35), time-varying menopausal status and hormone usage (premenopausal [reference], postmenopausal and never hormone therapy use, postmenopausal and past hormone therapy use, or current hormone therapy use), parity (≤1 [reference], 2, ≥3 births), regular aspirin use (yes, no [reference]), physical activity (0 [reference], 0.1-1.0, 1.1-3.4, 3.5-5.9, ≥6 hours/week), smoking status (never smoker [reference], former smoker, current smoker: 1-14, 15-24, ≥25 cigarettes/d), and Alternative Healthy Eating Index diet quality score (quintiles, with the lowest quintile [reference] representing the least healthy diet). Women who were oral contraceptive users (9899 participants) were excluded from all mediation analyses; women who developed type 2 diabetes (205 participants), chronic hypertension (4810 participants), or hypercholesterolemia (11 858 participants) before 1993 were additionally excluded from the analyses in which they were tested as a mediator. A total of 55 470 participants contributed to the joint mediation analysis.

^b^ Proportion of the association jointly mediated by type 2 diabetes, chronic hypertension, and hypercholesterolemia. Mediation analyses assume that there is no unmeasured exposure-outcome confounding, no unmeasured mediator-outcome confounding, no unmeasured exposure-mediator confounding, and no mediator-outcome confounder affected by exposure. The overall proportion mediated considers the proportion of the associations that is jointly accounted for by all 3 CVD risk factors (hypercholesterolemia, chronic hypertension, and type 2 diabetes).

To refine the exposure categories, we excluded women who reported no periods or women who reported that their periods were too irregular to estimate. To reduce potential reverse causation, we excluded women who received a diagnosis of cancer, type 2 diabetes, hypercholesterolemia, or chronic hypertension before 1993. To evaluate selection bias, we included previously excluded women who provided partial menstrual cycle characteristic information at ages 14 to 17 years (873 of 108 960 participants [0.8%]), 18 to 22 years (3235 of 108 960 participants [3.0%]), and 29 to 46 years (25 211 of 108 960 participants [23.1%]). To account for the role of BMI across the lifespan, we adjusted for time-varying BMI instead of baseline BMI. To assess how eating disorders may factor into our results, we excluded women with a BMI less than 18.5 at baseline or during follow-up. The associations of irregular and long menstrual cycles with a greater rate of CVD persisted across all sensitivity analyses (eTable 5 in the Supplement).

## Discussion

Over 24 years of follow-up, an increased rate of CVD was observed among women with greater menstrual cycle irregularity and longer menstrual cycle length in both early adulthood (ages 18 to 22 years) and mid-adulthood (29 to 46 years); similar trends were also observed for cycle characteristics in adolescence (14 to 17 years), but these were weaker than those during adulthood.

Consistent with prior literature, the association between cycle regularity and CVD appeared to be primarily driven by an increased rate of CHD events rather than stroke.^7,9^ An analysis of 82 439 NHS women found that those who reported usually irregular or very irregular cycles between ages 20 and 35 years had a 25% and 67% increased rate of CHD after 14 years of follow-up, respectively.^9^ This is similar to the 27% and 54% increased rates of CHD observed among women who reported usually irregular or always irregular or no period at ages 29 to 46 years with 24 years of follow-up in the current NHS II analysis, which incorporated contemporaneously reported cycle characteristics in more detail (across 3 stages of the reproductive lifespan) than the previous NHS analysis. Similarly, an analysis conducted among 15 005 women in the Kaiser Foundation Health Plan found an association between cycle irregularity and CHD mortality but not with cerebrovascular mortality.^7^ The current analysis provides longer follow-up (24 vs 17 years) in a larger population with information on behavioral factors (eg, diet and physical activity) and incident metabolic disorders, permitting more thorough control for confounding and allowing us to examine the portion of the association between cycle characteristics and CVD accounted for by the development of hypercholesteremia, chronic hypertension, and type 2 diabetes. Furthermore, we found that: (1) women who reported persistent cycle irregularity or persistent long cycle lengths across both early and mid-adulthood and (2) those whose cycle changed from regular during adolescence to irregular in mid-adulthood experienced the highest relative rates of CVD. These findings suggest that the transition of menstrual cycle phenotypes might be a surrogate for metabolic changes (eg, insulin resistance) that play a role in CVD development.

We observed an increased rate of CVD among women reporting oral contraceptive use only at ages 14 to 17 years, which might represent confounding by indications for oral contraceptive use (such as by polycystic ovary syndrome [PCOS] or endometriosis, which are also associated with future CVD risk).^27,28^ As oral contraceptives are more likely to be used solely for contraception during adulthood, they become weaker proxies for reproductive or gynecologic conditions, which may partly explain the null associations of oral contraceptive use at ages 18 to 22 and 29 to 46 years with CVD.

Unhealthy behaviors and obesity in childhood have been associated with menstrual irregularity and may affect cardiometabolic health across the life course through alterations in metabolism, fat storage, and body composition.^29,30^ PCOS, which is characterized by irregular cycles and ovulatory dysfunction, hyperandrogenism, and polycystic ovarian morphology,^27,31^ is the most common cause of irregular menstrual cycles. Approximately 90% of women with cycle irregularities or oligomenorrhea have clinical, laboratory, or ultrasound evidence of PCOS.^27,32,33^ A 2020 meta-analysis^34^ including 23 cohort studies showed that women with PCOS were at increased risk of cardiometabolic disease including CVD. Meanwhile, CVD risk factors, including dyslipidemia, type 2 diabetes, and abnormal vascular and endothelial function, are well described among women with PCOS.^11,27^ Therefore, the associations observed between cycle irregularity and long cycle length during early and mid-adulthood with CVD and the mediating role of hypercholesteremia, chronic hypertension, and type 2 diabetes are likely attributable to underlying PCOS.^27,32,33^ Finally, cycle dysfunction can also be indicative of endometriosis, depleted ovarian reserve, or disrupted hormonal environment (eg, hyperinsulinemia and hyperandrogenaemia), which are also associated with adverse cardiometabolic health.^1,35,36,37,38^

### Limitations

This study had several limitations. First, cycle characteristics were retrospectively reported for ages 14 to 17 and 18 to 22 years at NHS II enrollment in 1989 when participants were ages 25 to 42 years. However, our primary analysis relied on cycle characteristics contemporaneously reported by participants in 1993 (at ages 29 to 46 years). Furthermore, given that our analysis revealed associations at the extremes of both regularity and length, we can expect any misclassification of exposure to be less than that within the normal and more mild dysfunction exposure categories. Cycle characteristics were also reported prior to the development of CVD; therefore, any exposure misclassification would be nondifferential with respect to the outcome and bias toward the null. Second, since participants who died or reported a first CVD event as of 1993 were excluded from the analysis, it is possible that the observed results underestimated the increased rate of CVD associated with cycle irregularity and extreme length. Third, 23% of participants did not report their cycle characteristics at all ages assessed (14 to 17 years, 18 to 22 years, and 29 to 46 years) and were excluded from the primary analysis, which may have led to selection bias. However, similar results were observed when we included those women who provided partial information on cycle characteristics. Fourth, as the NHS II cohort includes primarily White non-Hispanic nurses, results may not be generalizable to more diverse populations with a different mixture of underlying cardiovascular risk factors. Fifth, although we conducted several sensitivity analyses to address potential sources of bias (eg, reverse causation, exposure misclassification, and selection bias), residual and unmeasured confounding (such as by undiagnosed or subclinical CVD risk factors and drug use) cannot be ruled out.

## Conclusions

In our prospective cohort study, irregular and long menstrual cycle lengths across the reproductive lifespan were associated with an increased risk of CVD later in life. Furthermore, we found that only a small proportion of the relation between cycle characteristics and CVD risk was driven by hypercholesterolemia, chronic hypertension, and type 2 diabetes. Our results suggest that menstrual cycle dysfunction may be a useful marker for identifying women who are more likely to develop CVD events later in life.

## Notes

Supplement. **eFigure.** Cohort Design, Data Collection, and Exclusion Criteria **eTable 1.** Age-standardized Characteristics of Included vs Excluded Women at Baseline in 1993 (Nurses’ Health Study II, 1993-2017) **eTable 2.** Multivariable Adjusted Hazard Ratios (HR) and 95% Confidence Intervals (CI) for Risk of Coronary Heart Disease (CHD) and Stroke According to Menstrual Cycle Regularity (at Ages 14-17 Years, 18-22 Years, and 18-48 Years) and Length (at Ages 18-22 Years and 29-46 Years) Among Premenopausal Women (n = 80 630; Nurses’ Health Study II, 1993-2017) **eTable 3.** Multivariable Adjusted Hazard Ratios (HR) and 95% Confidence Intervals (CI) for Cardiovascular Disease (CVD) Events According to Joint Exposure Categories of Menstrual Cycle Regularity and Length Among Premenopausal Women (n = 80 630; Nurses’ Health Study II, 1993-2017) **eTable 4.** Multivariable Adjusted Hazard Ratios (HR) and 95% Confidence Intervals (CI) for Cardiovascular Disease (CVD) Events According to Irregular and Long Menstrual Cycles at Ages 29-46 Years, Stratified by Behavioral Factors and Body Mass Index (n = 80 630; Nurses’ Health Study II, 1993-2017) **eTable 5.** Sensitivity Analyses for the Association of Menstrual Cycle Regularity (at Ages 14-17 Years, 18-22 Years, 29-46 Years) and Length (at Ages 18-22 Years and 29-46 Years) With CVD (Nurses’ Health Study II, 1993-2017)
