# Supplementary material for: Menstrual Cycle Regularity and Length Across the Reproductive Lifespan and Risk of Cardiovascular Disease
Source: JAMA Netw Open. 2022 Oct 25;5(10):e2238513. doi: 10.1001/jamanetworkopen.2022.38513 (PMC9597395; doi:10.1001/jamanetworkopen.2022.38513)
Supplement: Supplement. — eFigure. Cohort Design, Data Collection, and Exclusion Criteria eTable 1. Age-standardized Characteristics of Included vs Excluded Women at Baseline in 1993 (Nurses’ Health Study II, 1993-2017) eTable 2. Multivariable Adjusted Hazard Ratios (HR) and 95% Confidence Intervals (CI) for Risk of Coronary Heart Disease (CHD) and Stroke According to Menstrual Cycle Regularity (at Ages 14-17 Years, 18-22 Years, and 18-48 Years) and Length (at Ages 18-22 Years and 29-46 Years) Among Premenopausal Women (n = 80 630; Nurses’ Health Study II, 1993-2017) eTable 3. Multivariable Adjusted Hazard Ratios (HR) and 95% Confidence Intervals (CI) for Cardiovascular Disease (CVD) Events According to Joint Exposure Categories of Menstrual Cycle Regularity and Length Among Premenopausal Women (n = 80 630; Nurses’ Health Study II, 1993-2017) eTable 4. Multivariable Adjusted Hazard Ratios (HR) and 95% Confidence Intervals (CI) for Cardiovascular Disease (CVD) Events According to Irregular and Long Menstrual Cycles at Ages 29-46 Years, Stratified by Behavioral Factors and Body Mass Index (n = 80 630; Nurses’ Health Study II, 1993-2017) eTable 5. Sensitivity Analyses for the Association of Menstrual Cycle Regularity (at Ages 14-17 Years, 18-22 Years, 29-46 Years) and Length (at Ages 18-22 Years and 29-46 Years) With CVD (Nurses’ Health Study II, 1993-2017) [file jamanetwopen-e2238513-s001.pdf]

Supplemental Online Content

Wang YX, Stuart JJ, Rich-Edwards JW, et al. Menstrual cycle regularity and length across the reproductive lifespan and risk of cardiovascular disease. *JAMA Netw Open.* 2022;5(10):e2238513. doi:10.1001/jamanetworkopen.2022.38513

eFigure. Cohort Design, Data Collection, and Exclusion Criteria

eTable 1. Age-standardized Characteristics of Included vs Excluded Women at Baseline in 1993 (Nurses' Health Study II, 1993-2017)

eTable 2. Multivariable Adjusted Hazard Ratios (HR) and 95% Confidence Intervals (CI) for Risk of Coronary Heart Disease (CHD) and Stroke According to Menstrual Cycle Regularity (at Ages 14-17 Years, 18-22 Years, and 18-48 Years) and Length (at Ages 18-22 Years and 29-46 Years) Among Premenopausal Women (n = 80 630; Nurses' Health Study II, 1993-2017)

eTable 3. Multivariable Adjusted Hazard Ratios (HR) and 95% Confidence Intervals (CI) for Cardiovascular Disease (CVD) Events According to Joint Exposure Categories of Menstrual Cycle Regularity and Length Among Premenopausal Women (n = 80 630; Nurses' Health Study II, 1993-2017)

eTable 4. Multivariable Adjusted Hazard Ratios (HR) and 95% Confidence Intervals (CI) for Cardiovascular Disease (CVD) Events According to Irregular and Long Menstrual Cycles at Ages 29-46 Years, Stratified by Behavioral Factors and Body Mass Index (n = 80 630; Nurses' Health Study II, 1993-2017)

eTable 5. Sensitivity Analyses for the Association of Menstrual Cycle Regularity (at Ages 14-17 Years, 18-22 Years, 29-46 Years) and Length (at Ages 18-22 Years and 29-46 Years) With CVD (Nurses' Health Study II, 1993-2017)

This supplemental material has been provided by the authors to give readers additional information about their work.

© 2022 Wang YX et al. *JAMA Network Open.*

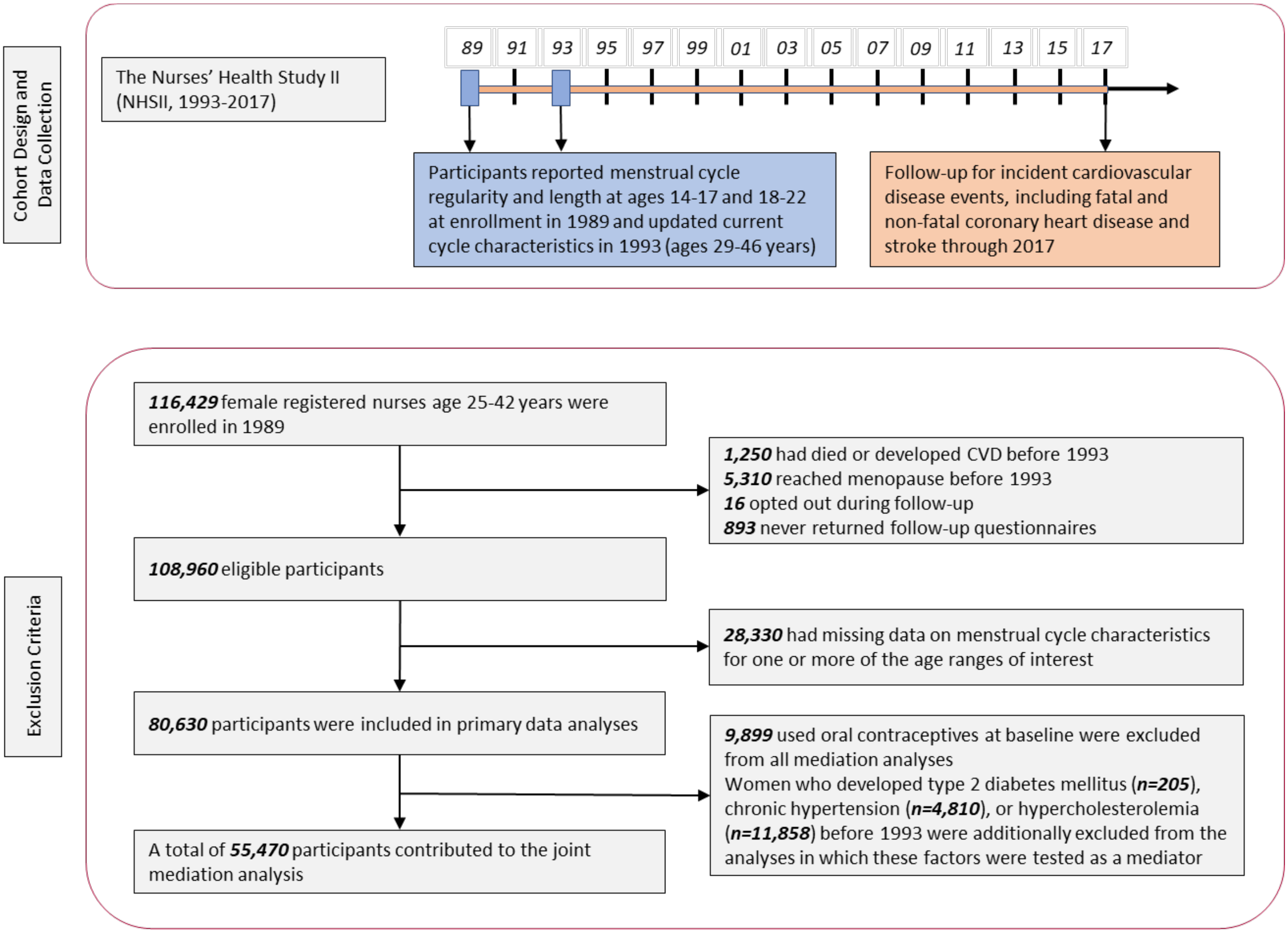

eFigure 1. Cohort design, data collection, and exclusion criteria

© 2022 Wang YX et al. *JAMA Network Open.*

eTable 1. Age-standardized characteristics of included versus excluded women at baseline in 1993 (Nurses' Health Study II, 1993-2017)

Characteristics (means [SD])[a,b]	Included[e] (n=80,630)	Excluded due to missing data on menstrual cycle characteristics[f] (n=28,330)
Age, y	37.7 (4.6)	38.8 (4.7)
Age at menarche, y	12.4 (1.4)	12.4 (1.5)
White[c]	77,108 (95.6)	25,898 (91.5)
Current smoker[c]	8442 (10.5)	3783 (13.3)
Physical activity, h/wk	2.7 (3.8)	2.9 (4.6)
Body mass index, kg/m^2	25.2 (5.6)	25.3 (5.6)
Regular aspirin use[c]	6845 (8.8)	1015 (10.9)
Alcohol consumption, g/d	3.2 (6.0)	3.1 (6.4)
Hypercholesterolemia[c]	13,340 (16.7)	4866 (16.5)
Chronic hypertension[c]	5537 (7.0)	2539 (8.4)
Type 2 diabetes mellitus[c]	232 (0.3)	116 (0.4)
Alternative Healthy Eating Index[d]	47.9 (10.8)	47.6 (10.7)
Lowest quintile (unhealthy)[c]	14,486 (17.8)	3409 (12.2)
Highest quintile (healthy)[c]	14,491 (18.1)	3333 (11.4)
Parity	1.7 (1.2)	1.6 (1.2)
Parental history of CVD before age 60[c]	13,333 (16.6)	4857 (16.8)

d: day; g: grams; h: hours; kg: kilograms; m: meters; SD: standard deviation; wk: week; y: years.

[a] Means (SD) for continuous variables and N (%) for categorical variables are standardized to the age distribution of the study population, except for age.

[b] A total of 244 (0.2%), 704 (0.6%), 260 (0.2%), 20,578 (18.9%), and 19,639 (18.0%) women had missing data on baseline smoking status, physical activity, BMI, aspirin use, and diet (including alcohol intake).

[c] N (%).

[d] The AHEI-2010 (Alternative Healthy Eating Index) score ranges from 0 (non-adherence) to 110 (perfect adherence) with a higher score indicating a healthier diet.

[e] Women with complete behavioral factors and menstrual cycle characteristics were included in our analysis.

[f] Women who were excluded from the analysis due to missing data on menstrual cycle characteristics at ages of 14-17, 18-22, and/or 29-46 years.

© 2022 Wang YX et al. *JAMA Network Open.*

eTable 2. Multivariable adjusted hazard ratios (HR) and 95% confidence intervals (CI) for risk of coronary heart disease (CHD) and stroke according to menstrual cycle regularity (at ages 14-17 years, 18-22 years, and 18-48 years) and length (at ages 18-22 years and 29-46 years) among premenopausal women (n=80,630; Nurses' Health Study II, 1993-2017)

Menstrual cycle characteristics	CHD			Stroke		
	Cases	Crude incidence per 1000 PY	HR (95% CI)[a]	Cases	Crude incidence per 1000 PY	HR (95% CI)[a]
**Cycle regularity**						
**14-17 years**						
Oral contraceptive users	120	0.69	1.26 (1.02, 1.55)	60	0.34	1.20 (0.90, 1.61)
Very regular	414	0.64	1 [Reference]	212	0.33	1 [Reference]
Regular	348	0.59	0.99 (0.86, 1.15)	195	0.33	1.05 (0.86, 1.28)
Usually irregular	154	0.58	1.01 (0.84, 1.22)	92	0.35	1.12 (0.88, 1.44)
Always irregular/no period	157	0.73	1.17 (0.97, 1.41)	77	0.36	1.10 (0.84, 1.43)
P for trend[b]			.19			.31
**18-22 years**						
Oral contraceptive users	789	0.68	1.16 (0.98, 1.38)	386	0.33	0.92 (0.74, 1.14)
Very regular	160	0.53	1 [Reference]	105	0.35	1 [Reference]
Regular	124	0.50	1.01 (0.80, 1.27)	89	0.36	1.07 (0.81, 1.42)
Usually irregular	62	0.58	1.19 (0.89, 1.60)	31	0.29	0.88 (0.59, 1.31)
Always irregular/no period	58	0.81	1.58 (1.17, 2.14)	25	0.35	1.01 (0.65, 1.56)
P for trend[b]			.01			.85
**29-46 years**						
Oral contraceptive users	86	0.37	1.00 (0.79, 1.27)	61	0.26	1.05 (0.79, 1.40)
Very regular	615	0.59	1 [Reference]	335	0.32	1 [Reference]
Regular	308	0.69	1.09 (0.95, 1.24)	156	0.35	1.02 (0.85, 1.24)
Usually irregular	106	0.99	1.27 (1.03, 1.57)	55	0.51	1.27 (0.95, 1.70)
Always irregular/no period	78	1.23	1.54 (1.21, 1.95)	29	0.46	1.11 (0.76, 1.63)
P for trend[b]			<.001			.20
**Cycle length**						
**18-22 years**						
Oral contraceptive users	789	0.68	1.18 (1.01, 1.36)	386	0.33	0.87 (0.72, 1.05)
≤25 days	34	0.53	1.02 (0.71, 1.46)	19	0.30	0.80 (0.50, 1.29)
26-31 days	242	0.51	1 [Reference]	170	0.36	1 [Reference]
32-39 days	72	0.55	1.18 (0.91, 1.53)	39	0.30	0.87 (0.62, 1.24)
≥40 days/too irregular to estimate	56	0.88	1.78 (1.33, 2.38)	22	0.34	0.96 (0.62, 1.50)
P for trend[b]			.002			.93
**29-46 years**						
Oral contraceptive users	86	0.37	0.98 (0.77, 1.23)	61	0.26	1.02 (0.77, 1.35)
≤25 days	218	0.79	1.06 (0.91, 1.23)	96	0.35	0.89 (0.71, 1.11)
26-31 days	685	0.61	1 [Reference]	379	0.34	1 [Reference]
32-39 days	108	0.60	1.06 (0.87, 1.31)	62	0.35	1.09 (0.83, 1.42)
≥40 days/too irregular to estimate	96	1.16	1.42 (1.14, 1.77)	38	0.46	1.06 (0.76, 1.49)
P for trend[b]			.05			.21

CI: confidence intervals; CHD: coronary heart disease; HR: hazard ratio; PY: person-years.

[a]Models were adjusted for age (continuous), age at menarche (continuous), race/ethnicity (White [ref], African-American, Hispanic, Asian), parental history of CVD before age 60 (yes, no [ref]), baseline body

© 2022 Wang YX et al. *JAMA Network Open.*

mass index (<23 [ref], 23-24.9, 25-29.9, 30-35, ≥35 kg/m^2), and time-varying menopausal status and hormone usage (premenopausal [ref], postmenopausal and never hormone therapy use, postmenopausal and past hormone therapy use, or current hormone therapy use), parity (≤1 [ref], 2, ≥3 births), regular aspirin use (yes, no), physical activity (0 [ref], 0.1-1.0, 1.0-3.5, 3.5-6.0, ≥6 hours/week), smoking status (never smoker [ref], former smoker, current smoker: 1-14, 15-24, ≥25 cigarettes/day), and Alternative Healthy Eating Index diet quality score (quintiles, with the lowest quintile [ref] representing the least healthy diet).

[b] *P*-value was estimated by excluding women using oral contraceptives.

© 2022 Wang YX et al. *JAMA Network Open.*

eTable 3. Multivariable adjusted hazard ratios (HR) and 95% confidence intervals (CI) for cardiovascular disease (CVD) events according to joint exposure categories of menstrual cycle regularity and length among premenopausal women (n=80,630; Nurses' Health Study II, 1993-2017)

Cycle regularity	Cycle length	CVD events	Crude incidence per 1000 PY	HR (95% CI)[a]
**18-22 years (early adulthood)**				
Oral contraceptive users		1,164	1.01	1.06 (0.95, 1.19)
Very regular or regular	<32 days	416	0.86	1 [Reference]
	≥32 days	60	0.91	1.15 (0.87, 1.50)
Irregular or no period	<32 days	47	0.91	1.08 (0.80, 1.46)
	≥32 days	129	1.01	1.24 (1.02, 1.51)
			p for multiplicative interaction	.94
			RERI (95% CI)	0.03 (-0.45, 0.51)
			p for additive interaction	.91
**29-46 years (mid-adulthood)**				
Oral contraceptive users		146	0.63	1.01 (0.84, 1.20)
Very regular or regular	<32 days	1,287	0.96	1 [Reference]
	≥32 days	118	0.81	0.96 (0.79, 1.16)
Irregular or no period	<32 days	83	1.52	1.16 (0.93, 1.45)
	≥32 days	182	1.57	1.35 (1.15, 1.58)
			p for multiplicative interaction	.24
			RERI (95% CI)	0.23 (-0.14, 0.59)
			p for additive interaction	.22

CI: confidence intervals; CVD: cardiovascular disease; HR: hazard ratio; RERI: relative excess risk due to interaction; PY: person-years.

[a]Models were adjusted for age (continuous), age at menarche (continuous), race/ethnicity (White [ref], African-American, Hispanic, Asian), parental history of CVD before age 60 (yes, no [ref]), baseline body mass index (<23 [ref], 23-24.9, 25-29.9, 30-35, ≥35 kg/m^2), and time-varying menopausal status and hormone usage (premenopausal [ref], postmenopausal and never hormone therapy use, postmenopausal and past hormone therapy use, or current hormone therapy use), parity (≤1 [ref], 2, ≥3 births), regular aspirin use (yes, no [ref]), physical activity (0 [ref], 0.1-1.0, 1.0-3.5, 3.5-6.0, ≥6 hours/week), smoking status (never smoker [ref], former smoker, current smoker: 1-14, 15-24, ≥25 cigarettes/day), and Alternative Healthy Eating Index diet quality score (quintiles, with the lowest quintile [ref] representing the least healthy diet).

© 2022 Wang YX et al. *JAMA Network Open.*

eTable 4. Multivariable adjusted hazard ratios (HR) and 95% confidence intervals (CI) for cardiovascular disease (CVD) events according to irregular and long menstrual cycles at ages 29-46 years, stratified by behavioral factors and body mass index (n=80,630; Nurses' Health Study II, 1993-2017)

**Behavioral factors and body mass index**	**CVD events**	**Cycle regularity[a] HR (95% CI)**		**Cycle length[a] HR (95% CI)**	
		**Very regular or regular**	**Irregular or no period**	**<32 days**	**≥32 days**
**Diet quality**					
Top 40%	517	1 [Reference]	1.33 (1.03, 1.71)	1 [Reference]	1.12 (0.88, 1.42)
Bottom 60%	1,153	1 [Reference]	1.27 (1.08, 1.49)	1 [Reference]	1.17 (1.00, 1.36)
p for multiplicative interaction		.78		.75	
RERI (95% CI)		0.02 (-0.38, 0.43)		0.10 (-0.24, 0.43)	
p for additive interaction		.91		.57	
**Smoking status**					
Never smokers	895	1 [Reference]	1.30 (1.08, 1.56)	1 [Reference]	1.13 (0.96, 1.34)
Current/ever smokers	775	1 [Reference]	1.32 (1.09, 1.61)	1 [Reference]	1.15 (0.95, 1.40)
p for multiplicative interaction		.90		.82	
RERI (95% CI)		0.17 (-0.25, 0.60)		0.03 (-0.34, 0.39)	
p for additive interaction		.43		.89	
**Physical activity**					
≥30 min/day	351	1 [Reference]	1.31 (0.96, 1.80)	1 [Reference]	1.18 (0.88, 1.57)
<30 min/day	1,319	1 [Reference]	1.28 (1.11, 1.49)	1 [Reference]	1.15 (1.00, 1.32)
p for multiplicative interaction		.82		.95	
RERI (95% CI)		-0.04 (-0.48, 0.41)		-0.003 (-0.37, 0.36)	
p for additive interaction		.88		.98	
**Body mass index**					
<25 kg/m^2	489	1 [Reference]	1.18 (0.88, 1.57)	1 [Reference]	1.23 (0.95, 1.58)
≥25 kg/m^2	1,181	1 [Reference]	1.43 (1.23, 1.66)	1 [Reference]	1.22 (1.05, 1.41)
p for multiplicative interaction		.18		.98	
RERI (95% CI)		0.55 (0.10, 1.01)		0.14 (-0.27, 0.55)	
p for additive interaction		.02		.49	

CI: confidence intervals; CVD: cardiovascular disease; HR: hazard ratio; kg: kilograms; m: meters; RERI: relative excess risk due to interaction; PY: person-years; Y: years.

[a]Models were adjusted for age (continuous), age at menarche (continuous), race/ethnicity (White [ref], African-American, Hispanic, Asian), parental history of CVD before age 60 (yes, no [ref]), baseline body mass index (<23 [ref], 23-24.9, 25-29.9, 30-35, ≥35 kg/m^2), and time-varying menopausal status and hormone usage (premenopausal [ref], postmenopausal and never hormone therapy use, postmenopausal and past hormone therapy use, or current hormone therapy use), parity (≤1 [ref], 2, ≥3 births), regular aspirin use (yes, no [ref]), physical activity (0 [ref], 0.1-1.0, 1.0-3.5, 3.5-6.0, ≥6 hours/week), smoking status (never smoker [ref], former smoker, current smoker: 1-14, 15-24, ≥25 cigarettes/day), and Alternative Healthy Eating Index diet quality score (quintiles, with the lowest quintile [ref] representing the least healthy diet), excluding the stratified variables.

eTable 5. Sensitivity analyses for the association of menstrual cycle regularity (at ages 14-17 years, 18-22 years, 29-46 years) and length (at ages 18-22 years and 29-46 years) with CVD (Nurses' Health Study II, 1993-2017)

Menstrual cycle characteristics	Excluding women reporting hypercholesterolemia as of 1993		Excluding women reporting "no period" or "irregular to estimate"		Excluding women reporting a history of cancer or diabetes as of 1993		Excluding women reporting chronic hypertension as of 1993		Including women reporting partial menstrual cycle data		Including time-varying BMI as a covariate		Excluding women with BMI<18.5 kg/m² at baseline or over follow-up	
	CVD events	HR (95% CI)[a]	CVD events	HR (95% CI)[a]	CVD events	HR (95% CI)[a]	CVD events	HR (95% CI)[a]	CVD events	HR (95% CI)[a]	CVD events	HR (95% CI)[b]	CVD events	HR (95% CI)[a]
**Cycle regularity**														
**14-17 years**														
Oral contraceptive users	128	1.27 (1.04, 1.55)	178	1.24 (1.04, 1.46)	170	1.22 (1.02, 1.45)	143	1.23 (1.01, 1.48)	252	1.19 (1.03, 1.37)	178	1.23 (1.04, 1.46)	177	1.25 (1.06, 1.49)
Very regular	429	1 [Reference]	622	1 [Reference]	599	1 [Reference]	486	1 [Reference]	842	1 [Reference]	622	1 [Reference]	616	1 [Reference]
Regular	384	1.02 (0.89, 1.17)	539	1.01 (0.90, 1.14)	510	0.99 (0.88, 1.12)	439	1.05 (0.92, 1.19)	752	1.04 (0.94, 1.15)	539	1.02 (0.91, 1.14)	521	0.99 (0.88, 1.11)
Usually irregular	170	1.05 (0.88, 1.26)	244	1.05 (0.90, 1.22)	230	1.02 (0.88, 1.19)	183	1.00 (0.84, 1.18)	337	1.08 (0.95, 1.22)	244	1.06 (0.91, 1.23)	239	1.05 (0.90, 1.21)
Always irregular/no period	158	1.16 (0.96, 1.39)	228	1.15 (0.99, 1.34)	216	1.11 (0.95, 1.30)	181	1.15 (0.97, 1.37)	323	1.17 (1.03, 1.33)	233	1.16 (1.00, 1.35)	231	1.16 (0.99, 1.35)
P for trend[c]	.15		.10		.27		.21		.02		.07		.08	
**18-22 years**														
Oral contraceptive users	799	1.06 (0.90, 1.25)	1,164	1.07 (0.93, 1.22)	1,104	1.05 (0.91, 1.20)	934	1.09 (0.94, 1.27)	1,593	1.07 (0.95, 1.21)	1,164	1.06 (0.93, 1.21)	1,144	1.07 (0.93, 1.22)
Very regular	187	1 [Reference]	263	1 [Reference]	256	1 [Reference]	206	1 [Reference]	345	1 [Reference]	263	1 [Reference]	259	1 [Reference]
Regular	161	1.09 (0.88, 1.34)	213	1.04 (0.87, 1.25)	201	1.01 (0.84, 1.22)	170	1.06 (0.87, 1.30)	282	1.05 (0.89, 1.22)	213	1.05 (0.87, 1.25)	209	1.04 (0.87, 1.25)
Usually irregular	71	1.18 (0.90, 1.55)	93	1.08 (0.85, 1.36)	86	1.03 (0.80, 1.31)	60	0.90 (0.67, 1.20)	125	1.10 (0.90, 1.35)	93	1.09 (0.86, 1.38)	90	1.07 (0.84, 1.36)
Always irregular/no period	51	1.26 (0.92, 1.72)	81	1.36 (1.06, 1.74)	78	1.33 (1.03, 1.71)	62	1.32 (1.00, 1.76)	110	1.35 (1.08, 1.67)	83	1.39 (1.09, 1.78)	82	1.38 (1.08, 1.77)
P for trend[c]	.13		.05		.11		.25		.02		.03		.04	
**29-46 years**														
Oral contraceptive users	102	1.00 (0.80, 1.24)	146	1.02 (0.85, 1.23)	136	0.98 (0.81, 1.18)	125	1.02 (0.84, 1.24)	154	1.02 (0.86, 1.22)	146	1.01 (0.84, 1.20)	142	1.03 (0.86, 1.24)
Very regular	686	1 [Reference]	945	1 [Reference]	912	1 [Reference]	775	1 [Reference]	990	1 [Reference]	945	1 [Reference]	926	1 [Reference]
Regular	318	1.04 (0.91, 1.18)	460	1.06 (0.95, 1.19)	433	1.04 (0.93, 1.16)	352	1.02 (0.89, 1.15)	479	1.05 (0.94, 1.17)	460	1.06 (0.95, 1.19)	452	1.07 (0.95, 1.19)
Usually irregular	98	1.20 (0.96, 1.48)	158	1.26 (1.06, 1.49)	148	1.23 (1.03, 1.47)	110	1.18 (0.97, 1.45)	162	1.22 (1.03, 1.45)	158	1.29 (1.08, 1.52)	157	1.27 (1.07, 1.51)
Always irregular/no period	65	1.42 (1.10, 1.84)	93	1.37 (1.10, 1.70)	96	1.34 (1.08, 1.66)	70	1.31 (1.02, 1.68)	258	1.33 (1.16, 1.54)	107	1.46 (1.20, 1.79)	107	1.43 (1.17, 1.75)
P for trend[c]	.007		<.001		.002		.02		<.001		<.001		<.001	
**Cycle length**														
**18-22 years**														
Oral contraceptive users	799	1.00 (0.87, 1.14)	1,164	1.05 (0.93, 1.17)	1,104	1.04 (0.92, 1.17)	934	1.08 (0.95, 1.23)	1,654	1.05 (0.95, 1.16)	1,164	1.04 (0.93, 1.16)	1,144	1.04 (0.93, 1.17)
≤25 days	39	0.93 (0.66, 1.29)	53	0.93 (0.70, 1.24)	51	0.93 (0.69, 1.24)	40	0.91 (0.66, 1.27)	72	0.87 (0.68, 1.11)	53	0.93 (0.70, 1.24)	50	0.89 (0.66, 1.20)
26-31 days	307	1 [Reference]	410	1 [Reference]	396	1 [Reference]	318	1 [Reference]	552	1 [Reference]	410	1 [Reference]	406	1 [Reference]
32-39 days	73	0.94 (0.73, 1.21)	111	1.06 (0.86, 1.30)	104	1.03 (0.83, 1.28)	87	1.07 (0.85, 1.36)	141	1.04 (0.86, 1.25)	111	1.06 (0.86, 1.31)	106	1.03 (0.83, 1.27)
≥40 days/too irregular to estimate	51	1.37 (1.02, 1.84)	33	1.34 (0.94, 1.91)	70	1.36 (1.05, 1.76)	53	1.28 (0.96, 1.72)	107	1.50 (1.22, 1.85)	78	1.48 (1.16, 1.89)	78	1.47 (1.16, 1.88)
P for trend[c]	.13		.16		.05		.07		<.001		.005		.006	
**29-46 years**														
Oral contraceptive users	102	0.99 (0.80, 1.23)	146	1.00 (0.84, 1.20)	136	0.96 (0.80, 1.16)	125	1.00 (0.82, 1.22)	154	1.00 (0.83, 1.19)	146	0.97 (0.81, 1.17)	142	1.00 (0.83, 1.20)
≤25 days	226	1.04 (0.90, 1.21)	313	1.00 (0.88, 1.14)	297	1.00 (0.87, 1.13)	235	0.95 (0.82, 1.10)	326	1.00 (0.88, 1.13)	313	0.99 (0.87, 1.12)	305	1.00 (0.87, 1.13)
26-31 days	745	1 [Reference]	1,057	1 [Reference]	1012	1 [Reference]	853	1 [Reference]	1,100	1 [Reference]	1,057	1 [Reference]	1,040	1 [Reference]
32-39 days	118	1.12 (0.92, 1.36)	166	1.05 (0.89, 1.24)	158	1.06 (0.89, 1.25)	129	1.04 (0.86, 1.25)	174	1.06 (0.91, 1.25)	166	1.08 (0.91, 1.27)	163	1.06 (0.89, 1.25)
≥40 days/too irregular to estimate	78	1.28 (1.01, 1.62)	80	1.33 (1.05, 1.67)	122	1.27 (1.05, 1.54)	90	1.26 (1.01, 1.57)	138	1.30 (1.08, 1.55)	134	1.36 (1.13, 1.63)	134	1.32 (1.10, 1.59)
P for trend[c]	.15		.09		.04		.04		.02		.005		.02	

[a]Models were adjusted for age (continuous), age at menarche (continuous), race/ethnicity (White [ref], African-American, Hispanic, Asian), parental history of CVD before age 60 (yes, no [ref]), baseline body mass index (<23 [ref], 23-24.9, 25-29.9, 30-35, ≥35 kg/m²), and time-varying menopausal status and hormone usage (premenopausal [ref], postmenopausal and never hormone therapy use, postmenopausal and past hormone therapy use, or current hormone therapy use), parity (≤1 [ref], 2, ≥3 births), regular aspirin use (yes, no [ref]), physical activity (0 [ref], 0.1-

© 2022 Wang Y et al. *JAMA Network Open.*

1.0, 1.0-3.5, 3.5-6.0, ≥6 hours/week), smoking status (never smoker [ref], former smoker, current smoker: 1-14, 15-24, ≥25 cigarettes/day), and Alternative Healthy Eating Index diet quality score (quintiles, with the lowest quintile [ref] representing the least healthy diet).
[b]Models were adjusted for age (continuous), age at menarche (continuous), race/ethnicity (White [ref], African-American, Hispanic, Asian), parental history of CVD before age 60 years (yes, no [ref]), and time-varying menopausal status and hormone usage (premenopausal [ref], postmenopausal and never hormone therapy use, postmenopausal and past hormone therapy use, or current hormone therapy use), parity (≤1 [ref], 2, ≥3 births), regular aspirin use (yes, no [ref]), body mass index (<23 [ref], 23-24.9, 25-29.9, 30-35, ≥35 kg/m^2), physical activity (0 [ref], 0.1-1.0, 1.0-3.5, 3.5-6.0, ≥6 hours/week), smoking status (never smoker [ref], former smoker, current smoker: 1-14, 15-24, ≥25 cigarettes/day), and Alternative Healthy Eating Index diet quality score (quintiles, with the lowest quintile [ref] representing the least healthy diet).
[c]*P*-value was estimated by excluding women using oral contraceptives.
